# Cabozantinib for HCC Treatment, From Clinical Back to Experimental Models

**DOI:** 10.3389/fonc.2021.756672

**Published:** 2021-10-13

**Authors:** Shanshan Deng, Antonio Solinas, Diego F. Calvisi

**Affiliations:** ^1^ Department of Bioengineering and Therapeutic Sciences and Liver Center, University of California, San Francisco, San Francisco, CA, United States; ^2^ Department of Biomedical Sciences, University of Sassari, Sassari, Italy; ^3^ Institute of Pathology, University of Regensburg, Regensburg, Germany

**Keywords:** hepatocellular carcinoma, multikinase inhibitor, cabozantinib, combination therapy, clinically, preclinical, c-MET

## Abstract

Hepatocellular carcinoma (HCC) is the fourth leading cause of cancer-related mortality worldwide. Patients with early-stage HCC can be treated successfully with surgical resection or liver transplantation. However, the usual late diagnosis of HCC precludes curative treatments, and systemic therapies are the only viable option for inoperable patients. Sorafenib, an orally available multikinase inhibitor, is a systemic therapy approved for treating patients with advanced HCC yet providing limited benefits. Consequently, new drugs have been developed to overcome sorafenib resistance and improve patients’ prognoses. A new promising strategy is using c-MET inhibitors, such as cabozantinib, as activation of c-MET occurs in up to 40% of HCC patients. In particular, cabozantinib, in combination with the checkpoint inhibitor atezolizumab, is currently in phase 3 clinical trial for HCC, and the results are eagerly awaited. Herein, we summarize and review the drugs approved for the treatment of advanced HCC, mainly focusing on the clinical and preclinical efficacy evaluation of cabozantinib. Also, we report the available preclinical data on cabozantinib-based combination therapies for HCC, current obstacles for cabozantinib therapy, and the future directions for cabozantinib-based treatment for HCC.

## Introduction

### Hepatocellular Carcinoma: A Deadly Tumor With Slowly Increasing Therapeutic Options

**Figure 1:**
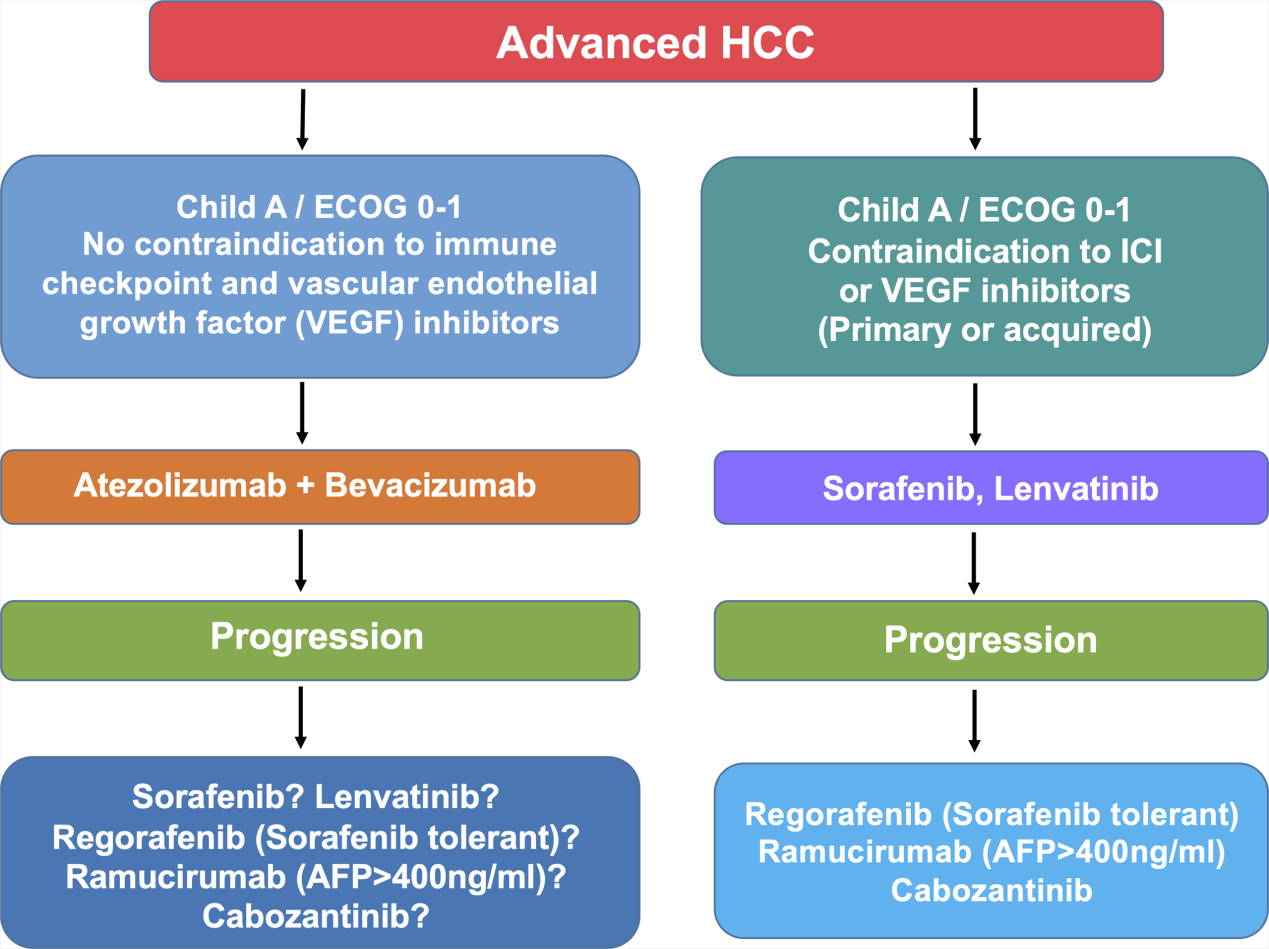
Algorithm illustrating the current treatment options (beyond the clinical trials) for patients with advanced HCC. Note that it remains unclear whether or not patients progressing on atezolizumab and bevacizumab benefit from second-line drugs tested such as sorafenib, lenvatinib, ramucirumab, and cabozantinib. Therefore, patients who develop adverse effects leading to the withdrawal of atezolizumab and bevacizumab should be offered the available sequence of targeted therapies.

Hepatocellular carcinoma (HCC) is the fourth cause of cancer-related deaths and the primary cause of death in patients with compensated cirrhosis (1). Standard-of-care includes hepatic resection, liver transplantation and local ablation in the early stages, chemoembolization in the intermediate stage, and systemic therapy in the advanced stage (2). Surveillance and allocation to treatment according to disease stage and liver dysfunction have led to significant improvement in the management of these patients. Also, the advent of new effective agents and the recent insights into the molecular classification of HCC open new scenarios. It is conceivable that current unmet needs such as the rate of recurrence after resection, and the survival time in patients with advanced disease will benefit from the administration of novel combined therapies. The armamentarium for treatment of advanced HCC has expanded and currently consists of sorafenib, lenvatinib, and the combination of atezolizumab and bevacizumab in the frontline and regorafenib, ramucirumab and cabozantinib, nivolumab, and pembrolizumab in the second and third line (**Figure 1**). This implies that the proper choice for each patient should consider the features of the patients, the efficacy and the toxicity of the therapeutic regimen, as well as the biology of the tumor. We review here the drugs approved for advanced HCC and focus on the clinical and molecular features of cabozantinib.

In 2008, sorafenib, a multikinase inhibitor active against vascular endothelial growth factor receptor (VEGFR) 2 and 3, fms like tyrosine kinase (FLT)3, KIT, platelet-derived growth factor receptor (PDGFR)β, RAF, BRAF WT, and BRAF V600E, was the first approved agent for advanced HCC. The phase III SHARP trial showed that sorafenib conferred survival improvement (10.7 months in the treatment group vs. 7.9 in the placebo group, hazard ratio (HR), 0.69; *p* < 0.001) and higher disease control rate (patients with complete response, partial response, and stable disease for a period > 4 weeks, 43% vs. 32%; *p* < 0.001) (3). The most frequent adverse effects of sorafenib were hand-foot skin reaction, diarrhea, and hypertension. Progression on therapy was related to primary and acquired resistance. Mechanisms of resistance to sorafenib have been elucidated only in part. Among them, hypoxia-induced factors, overproduction of vascular endothelial growth factor (VEGF), and inhibition of the RAF/MEK/ERK pathway result in the activation of EGFR, AKT/TOR, and MET proto-oncogene (c-MET)/hepatocyte growth factor axes. Therefore, strategies to overcome sorafenib and other tyrosine kinase inhibitors are crucial in developing effective therapies. Patterns of disease progression on sorafenib are also relevant, as the survival probability progressively decreases in patients with an increase of the tumor characterized by intrahepatic lesions < 20%; intrahepatic lesions > 20%; extrahepatic lesions ≤ 20%; new extrahepatic lesions and/or vascular invasion (4).

Lenvatinib, a multikinase inhibitor active on VEGFR 1-3, fibroblast growth factor receptor (FGFR), KIT, RET, and PDGFR, was demonstrated not inferior to sorafenib in phase III multicenter trial (5). Enrolled patients had a liver involvement < 50% and were free of main portal invasion. Median overall survival was 13.6 months vs 12.3 (HR, 0.92; 95% confidence interval (CI), 0.79-1.06). The surrogate endpoint progression-free survival (PFS) was significantly longer in the lenvatinib group (HR, 0.65; 95% CI, 0.56-0.77; *p* < 0.001). The adverse effects associated with lenvatinib were hypertension and hand and foot skin reaction.

The last year has witnessed a paradigm shift in the frontline treatment of advanced hepatocellular carcinoma when the combination of atezolizumab and bevacizumab was approved for the treatment of newly diagnosed patients with advanced hepatocellular carcinoma. Atezolizumab is a monoclonal antibody targeting the protein programmed cell death ligand (PD-L)-1 and thereby enhancing the immune response against tumor cells. Bevacizumab is a monoclonal antibody against VEGF. The approval of this regimen is based on the results of the IMbrave150 phase III trial (6). Patients treated with atezolizumab and bevacizumab showed better overall survival (HR, 0.58; 95% CI, 0.42-0.79; *p* < 0.001) and PFR (HR, 0.59; 95% CI, 0.57-0.76) than those treated with sorafenib. Objective response rate (ORR) was found in 27.3% of 326 patients treated with the combination vs. 11.9% of 159 treated with sorafenib. Eighteen patients in the combination group vs. no patient in the sorafenib group experienced a complete response. The median time to deterioration of the quality of life was 11.2 months with atezolizumab plus bevacizumab vs. 3.6 with sorafenib. Adverse events occurred in 56.5% vs. 55.1%. Hypertension and proteinuria were the most frequent side effects in those treated with atezolizumab and bevacizumab.

Among the second-line agents, regorafenib was the first effective systemic treatment in patients who progressed on sorafenib therapy. Regorafenib is a multikinase inhibitor with activity on VEGFR 1-3, KIT, RET, PDGFRβ, RAF, BRAF WT, and BRAF V600E. Patients who received regorafenib in the context of the controlled phase 3 trial RESORCE showed an improved overall survival (HR, 0.63; 95% CI, 0.50-0.79; *p* < 0.0001), an improved PFR (HR, 0.46; 95% CI, 0.37-0.56; *p* < 0.0001) (7). The median survival time was 10.6 months (95% CI, 9.1-12.1) with regorafenib and 7.8 (95% CI, 6.3-8.8) with placebo. Two patients in the regorafenib group vs. no patient in the placebo group experienced a complete response by mRECIST. Common adverse events were hypertension (15% of treated patients), hand-foot skin reaction, diarrhea, and fatigue. Importantly, the study was balanced regarding the pattern of progression of the disease on sorafenib. The retrospective analysis of the RESORCE trial aimed to evaluate the efficacy of the sequential treatment showed that the median survival time from the start of prior sorafenib was 26 months in the regorafenib group and 19.2 months in the placebo group (8).

Ramucirumab is a monoclonal antibody active against VEGFR2. In the controlled phase 3 (REACH) trial, ramucirumab did not improve the overall survival, when compared to placebo, in which patients with advanced HCC that progressed on sorafenib or were intolerant to sorafenib. However, a significant benefit was observed in the subgroup of patients with baseline alpha-fetoprotein ≥ 400 ng/mL. This finding was confirmed in the phase 3 REACH2 trial, in which all enrolled patients had alpha-fetoprotein concentrations ≥ of 400 ng/mL (9). The median survival time was 8.5 months (95% CI, 7.0-10.6) in the experimental group vs 7.3 months (5.4-9.1) in the placebo group (HR, 0.71; 95% CI, 0.531-0.949; *p* = 0.0199). ORR did not differ significantly in the two groups. Side effects were hypertension and hyponatremia. Three patients in the treatment group died of renal failure.

Pembrolizumab is a monoclonal humanized IgG4/K antibody active against PD-1. In phase 2 non-randomized open-label trial (Keynote 224), the drug showed promising activity in patients previously treated with sorafenib. Disease control was obtained in 64 of 104 patients (10). A subsequent phase III trial (Keynote 240) did not reach the pre-specified criteria of significance (11). However, the median survival was 13.9 months in the pembrolizumab group vs. 10.6 months in the best supportive care group. Adverse effects leading to discontinuation of treatment were reported in 17% of patients.

Nivolumab is a fully human monoclonal IgG4 antibody active against PD-1. The open-label phase 2 trial Check-Mate 040 showed an ORR of 15% in the dose-escalation phase (12). The disease control rate was 58% (95% CI, 43-72). The median duration of response was 17 months (95% CI, 6-24). Both pembrolizumab and nivolumab have been approved by FDA for patients with advanced HCC. However, in July 2021, Bristol Myers Squibb voluntarily withdrew the indication for nivolumab as a single agent for patients with HCC who were previously treated with sorafenib. This decision was based on the unmet post-marketing requirements of a confirmatory benefit.

Cabozantinib is a multikinase inhibitor of VEGFR2, c-MET, AXL receptor tyrosine kinase (AXL), FLT3, c-KIT, and c-RET. The properties of cabozantinib are discussed in detail in the second part of this review. Cabozantinib was approved in 2019 for patients with advanced hepatocellular carcinoma who had been previously treated with sorafenib. This approval was based on the results of phase 3 CELESTIAL trial (13). All 750 patients included in the study had progressed or had experienced significant toxicity during the prior treatment, and extrahepatic spread of the disease was reported in 78% of the cases. The median overall survival was 10.2 months (95% CI, 9.1-12) in the cabozantinib group vs 8 months (95% CI, 6.8-9.4) in the placebo group (HR, 0.76; 95% CI, 0.63-0.92; *p* = 0.005). The median PFS was 5.2 months with cabozantinib and 1.9 with placebo (HR, 0.44; 95% CI, 0.36-0.52; *p* < 0.001). Side effects were hand-foot skin reaction, hypertension, increased aspartate aminotransferase levels, fatigue, and diarrhea. These results were recently confirmed by two real-life cohorts of patients with advanced HCC treated with cabozantinib (14, 15). In 88 patients from Austria, Switzerland, and Germany, partial response was documented in 6%, stable disease in 32%, and progressive disease in 32%. The overall survival was 9.7 months in Child-Pugh A patients and 3.4 months in Child-Pugh B patients (14). In 96 Child-Pugh A patients from Italy, the median overall survival was 12.1 months, and progression-free survival was 5.1 months (15).

In 2020, a matching-adjusted indirect comparison (MAIC) was carried out to compare the efficacies and safety profiles of cabozantinib and regorafenib by indirectly comparing the results of the CELESTIAL and RESORCE trials in patients who received first-line sorafenib only (16). Results showed that compared with regorafenib, cabozantinib could achieve similar overall survival and longer PFS in advanced HCC patients who progressed after receiving sorafenib treatment. Another similar MAIC study compared the efficacies and treatment-related adverse events of cabozantinib in the CELESTIAL trial and ramucirumab in the REACH2 trial (17). Cabozantinib achieved a nonsignificant overall survival as ramucirumab but significantly prolonged PFS (5.5 [4.6-7.4] months vs. 2.8 [2.7-4.1] months; *p* = 0.016) in HCC patients with serum alpha-fetoprotein over 400 ng/mL. A recent phase 2 multicenter study (Trial ID: NCT03586973) conducted earlier this year in Japanese patients with advanced HCC who have received one or two lines of systemic therapy (sorafenib included) showed that cabozantinib at a dose of 60 mg/day had excellent efficacy and safety profile (18). Altogether, cabozantinib shows great promise in treating HCC in the clinic as a second-line of treatment.

In June 2021, Exelis and Ipsen, the manufacturer of cabozantinib, reported the interim analysis of this trial. In combination with atezolizumab, cabozantinib significantly reduced the risk of disease progression or death by 37% compared with sorafenib (hazard ratio, 0.63; 99% CI, 0.44-0.91; *p* = 0.0012) (https://www.businesswire.com/news/home/20210627005058/en/). The analysis of the overall survival showed a trend favoring the combination of cabozantinib and atezolizumab but did not reach statistical significance. Therefore, the future of cabozantinib development as first-line therapy for HCC is uncertain. Due to the efficacious anticancer properties of cabozantinib, the combination use based on cabozantinib is under intense investigation. Indeed, a phase 1/2 clinical trial study (Trial ID: NCT03539822) is currently evaluating cabozantinib’s safety, tolerability, and efficacy combined with the PD-L1 inhibitor durvalumab in patients with gastrointestinal malignancies. The results are eagerly awaited (19). Another COSMIC-312 phase 3 study (Trial ID: NCT03755791) is also carrying out to test the safety and efficacy of the combination of cabozantinib and atezolizumab as a first-line treatment in adult patients with advanced HCC who have not received systemic anticancer therapy before versus the standard treatment regimen sorafenib (20). Scanty data are available on the efficacy of the combination of nivolumab, ipilimumab, and cabozantinib. The disease control rate was 83% in 35 patients treated with nivolumab, ipilimumab, and cabozantinib, and 81% in 36 patients treated with nivolumab and cabozantinib, respectively (21). Because the ORR of nivolumab monotherapy in patients who received nivolumab monotherapy (3 mg/kg) during the dose-expansion phase was 20% (95% CI, 15-26), and 15% (95% CI, 6-28) during dose escalation (12), the ORR of the combination of cabozantinib and nivolumab was 17% (21), the ORR of the combination of nivolumab and ipilimumab was greater than 30% with overall high patient survival rate and manageable safety profiles (22), the combination of cabozantinib and immunotherapy seems not to have strong synergistic anticancer efficacy, and cabozantinib may not be ideal for immunotherapy-based combination.

The occurrence and progression of HCC are always associated with dysregulation of various cellular mechanisms (e.g., proliferation, survival, angiogenesis, etc.) caused by virus infection, alcoholism, metabolic diseases, and carcinogen exposure (23). Drugs currently used for the treatment of HCC could inhibit these cellular processes by targeting multiple signaling pathways. Among all the available therapies, sorafenib, lenvatinib, and the combination of atezolizumab and bevacizumab are the first-line systemic therapy of HCC, and regorafenib, ramucirumab, and cabozantinib are second-line systemic therapy (**Table 1**). As an oral tyrosine kinase inhibitor that can inhibit the growth of multiple tumors by inhibiting various receptor tyrosine kinases (RTKs) (e.g., VEGFR2, c-MET), cabozantinib is approved for the treatment of patients with HCC that have been previously treated with sorafenib. From the MAIC and another similar MAIC study comparing the efficacy and safety profiles of cabozantinib with other systemic therapies (regorafenib and ramucirumab), cabozantinib achieved a similar overall survival as regorafenib or ramucirumab but significantly prolonged the progression-free survival of corresponding patients with HCC (16, 17). In addition, cabozantinib is the only drug given to all HCC patients after first-line systemic treatment (e.g., sorafenib) regardless of the baseline AFP level or the tolerability of sorafenib treatment of patients, which further distinguishes cabozantinib from ramucirumab and regorafenib (14). Furthermore, as people’s understanding of the tumor immune microenvironment in HCC continues to increase, the development of immunotherapy alone or in combination with targeted therapy has become a hot topic for the treatment of HCC. By targeting multiple RTKs, cabozantinib shows an immunomodulatory effect on the tumor microenvironment and makes tumor cells more sensitive to immune-mediated killing (24, 25). Overall, cabozantinib represents a promising drug for treating HCC as a first-line or immunotherapy-based combination therapy.

**Table 1:** Overall survival and median survival periods from 6 phase 3 trials in which overall survival was the primary end-point of efficacy.

YEAR	DRUG	ECOG-PERFORMANCE STATUS	CHILD-PUGH GRADE	OVERALL SURVIVAL (hazard ratio)	MONTHS OF SURVIVAL (median)	LINE OF THERAPY
2007	SORAFENIB	≤ 2	A	0.69 (*vs.*. placebo)	10.7 *vs.* 7.9	1
2018	LENVATINIB	≤ 1	A	0.92 (*vs.* sorafenib)	13.6 *vs.* 12.3	1
2020	ATEZOLIZUMAB AND BEVACIZUMAB	≤ 1	A	0.58 (*vs.* sorafenib)	19.2 *vs.* 13.4	1
2017	REGORAFENIB	≤ 1	A	0.63 (*vs.* placebo)	10.6 *vs.* 7.8	2
2019	RAMUCIRUMAB	≤ 1	A	0.71 (*vs.* placebo)	8.5 *vs.* 7.3	2
2019	CABOZANTINIB	≤ 1	A	0.76 (*vs.* placebo)	10.2 *vs.* 8.0	2

Since the clinical trials of cabozantinib-based combinations are ongoing, at this stage, however, we should go back to the preclinical studies to find more evidence that supports the use of the cabozantinib-based combinations. Thus, in the following sections, we will mainly focus on the recent advances of cabozantinib either in monotherapy or synergistic with other drugs for treating HCC in preclinical studies.

## Molecular Mechanisms of Cabozantinib in Cancer Treatment

Receptor tyrosine kinases are critical regulatory signaling proteins involved in many cellular processes, including cell growth, division, survival, and migration (26, 27). Dysregulation of RTK signaling has been linked to cancer formation, progression, and metastasis. RTKs are therefore druggable targets for cancer therapy (28, 29). Cabozantinib, also known as the brand name Cabometyx, is an orally small tyrosine kinase inhibitor (TKI). It was developed by Exelixis and can target the c-MET and VEGFR2 with potent efficacy. It is also active against other RTKs, such as AXL, c-RET, c-KIT, and FLT3 (30–32). All these receptor tyrosine kinases have been reported to be involved in cancer development and progression. Cabozantinib functions by inhibiting the activities of the abovementioned multiple tyrosine kinases and preventing receptor phosphorylation and subsequent signal transduction. This leads to cancer cell apoptosis, reduced proliferation and metastasis, and decreased tumor angiogenesis, ultimately resulting in tumor regression (33, 34). For TKIs that specifically target one receptor, their therapeutic strength may be limited by the insufficient efficacy and rapid emergence of resistant clones (35, 36). Multi-tyrosine kinases inhibitors, such as cabozantinib, have the advantage of simultaneously targeting several key signaling pathways in tumor cells, providing more substantial anti-tumor activities and potentially preventing the occurrence of resistant clones.

Since c-MET, VEGFR, and AXL proteins are the three main cabozantinib targets for cancer therapy, these RTKs and how cabozantinib inhibits their activities are reported below.

### c-MET Pathway

c-MET is a tyrosine kinase receptor that is mainly expressed in epithelial cells. c-MET binds to its ligand, hepatocyte growth factor (HGF), leading to the receptor homodimerization, phosphorylation of tyrosine residues, and recruitment of the downstream signaling effectors, such as phosphatidylinositol 3-kinase (PI3K) (37, 38). Thus, in normal conditions, c-MET activation, *via* binding to HGF, promotes epithelial cell growth. The pathway has a pivotal role in regulating embryonic development, organogenesis, and regeneration of damaged tissues in adulthood (39). Overexpression, alteration, or mutations of c-MET have been identified in multiple tumor types. Indeed, in cancers, the HGF/c-MET cascade is closely related to the proliferation, differentiation, cytoskeleton rearrangement, apoptosis resistance, and invasion of tumor cells, making c-MET a target for anticancer therapy (32, 40, 41). Cabozantinib blocks c-MET phosphorylation and inhibits its transduction by non-selectively binding to c-MET as an ATP competitor. This leads to the suppression of cancer cell survival, proliferation, invasion, or even metastasis (42).

### VEGFR Pathway

VEGF is an essential protein for the growth and differentiation of endothelial cells. Specifically, it is involved in the formation of blood vessels in the embryo (known as vasculogenesis) and the development of the new blood vessels in adult tissues (known as angiogenesis) (43, 44). VEGFRs are the receptors of VEGF. There are three major isoforms of VEGFRs: VEGFR1, VEGFR2, and VEGFR3. Like c-MET, all VEGFR isoforms bind to VEGFs on the cell surface, causing dimerization, subsequent autophosphorylation, and initialization of downstream signaling pathways. These processes require additional modulators, such as heparan sulfate, neuropilins, or integrins (45, 46). Once activated, VEGFR1 functions to negatively regulate VEGFR2 and the migration of monocytes and macrophages. VEGFR2 activation triggers multiple cellular processes of VEGF, including the differentiation of vascular endothelial progenitor cells, vascular permeability, angiogenesis and endothelial cell migration, proliferation, and survival. Therefore, VEGFR2 is the major isoform that regulates angiogenesis in tumor tissues. VEGFR3 instead primarily modulates lymphangiogenesis (47–49).

Nutrients and oxygen are required for tumor growth. The development of new blood vessels, formed by the process of angiogenesis, can provide these essential elements (50). Overexpression of VEGF has been reported in many cancers, including colon, lung, and other tumors, as an essential regulator of angiogenesis. VEGF is commonly induced by cancer hypoxia due to the fast growth of the tumor and the limited number of blood vessels (49, 51). Since VEGF and VEGFR are necessary for the development and metastasis of many tumors, targeted treatments against VEGR or VEGFR can be used as an effective anticancer therapy. The mechanism of action of cabozantinib to interact with VEGFR is the same as c-MET, and the inhibition of VEGFR leads to the suppression of angiogenesis in cancer tissues.

### AXL Pathway

Another primary target of cabozantinib is the AXL protein, a cell surface RTK that belongs to the TAM family, which includes TYRO3, ​​AXL, and MERTK. The ligands of AXL are the growth arrest-specific 6 (GAS6) and protein S (52). By binding to these ligands, the AXL receptor undergoes dimerization, autophosphorylation, transphosphorylation, and activation of downstream signaling pathways, such as the PI3K/AKT/mTOR and MEK/ERK cascades (53, 54). Through the transduction of these signals, AXL regulates cell proliferation, survival, epithelial-mesenchymal transition, invasion, migration, and immune function (53, 55). Clinically, the increased expression of AXL is associated with augmented invasiveness and metastasis of many tumors, including breast, lung, pancreatic, ovarian, and colon cancer. Besides, high levels of AXL1 have been linked to poor prognosis and survival outcomes (56–58). Cabozantinib inhibits AXL tyrosine kinase activity and blocks the activation of downstream pathways for cancer therapy (59).

**Figure 2:**
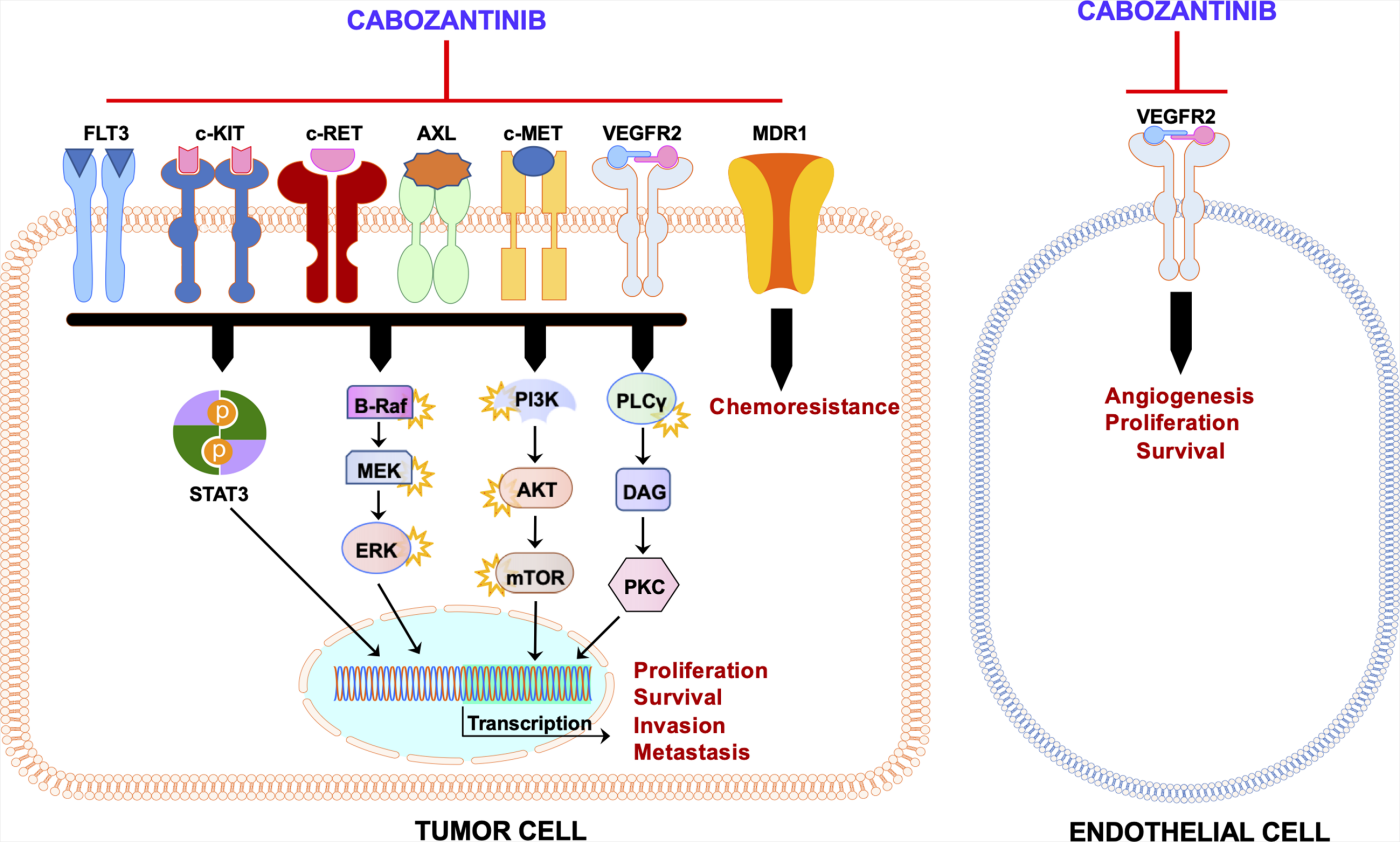
Schematic representation of the mechanism of action of cabozantinib. Administration of cabozantinib to cancer cells leads to the inhibition of several tyrosine kinases (c-MET, VEGFR2, AXL, c-KIT, c-RET, and FLT3) as well as MDR1/P-glycoprotein in the tumor cell and/or in the endothelial cell. These tyrosine kinases, once activated, induce a plethora of downstream pathways (STAT3, Ras/BRAF/MEK/ERK, AKT/mTOR, PLCγ/PKC, etc.) that lead to several biologic effects on the cells (proliferation, survival, migration, chemoresistance, etc.). These activities are blunted by cabozantinib. Black arrows indicate activation, whereas red blunted arrows indicate inhibition.

Thus, cabozantinib exerts its clinical activity presumably by suppressing c-MET, VEGFR2, and AXL concomitantly. In addition to c-MET, VEGFR, and AXL, cabozantinib targets and inhibits other tyrosine kinases, including RET, KIT, and FLT3, also involved in tumorigenesis (**Figure 2**). Therefore, cabozantinib is a potent anti-tumor drug due to its ability to target multiple pathways in cancer cells, leading to decreased tumor growth and angiogenesis and eventually tumor regression.

## Cabozantinib Monotherapy and HCC Treatment

Cabozantinib was first approved by the U.S. Food and Drug Administration (FDA) as a first-line treatment for medullary thyroid cancer in 2012. Cabozantinib was extended to be used as a second-line treatment of kidney cancer in 2016. Currently, Cabozantinib has been investigated to treat various malignancies, including lung cancer, prostate cancer, neuroblastoma, and HCC, in clinical trials (32, 60, 61).

Overexpression or activation of c-MET has been observed in up to 40% of HCC patients. HCC is also known to be hyper-vascular, indicating increased angiogenesis in this tumor type. Cabozantinib, due to its property of targeting multiple pathways related to HCC development and progression, represents a promising drug for the treatment of this deadly malignancy (62).

Many studies have reported the efficacy of cabozantinib on HCC *in vitro* and *in vivo*. In 2014, Xiang et al. showed that cabozantinib could inhibit the proliferation, migration, and invasion of p-MET-overexpressing HCC cells by suppressing the activity of MET and its downstream effectors, including STAT3, AKT, and ERK1/2, at nM concentrations (63). Moreover, cabozantinib treatment hampered the growth of HCC tumors by repressing angiogenesis, inhibiting proliferation, and inducing apoptosis and metastasis *in vivo* using xenograft models (63). Importantly, it was shown that cabozantinib efficacy was more pronounced on HCC xenograft models characterized by activation of c-MET (63). Recently, Caruso’s group conducted a screening experiment to evaluate the effectiveness of 31 drugs approved by the FDA or under clinical investigations for treating HCC using a panel of liver cancer cell lines (64). The results indicated that all multikinase inhibitors, including cabozantinib, had overall weak efficacy over 34 liver cancer cell lines. However, cabozantinib was efficacious on liver cancer cell lines expressing epithelial features genes of hepatocyte and liver progenitor markers. In particular, cabozantinib was highly cytotoxic on c-MET-amplified cell lines (64). These findings suggest that the anti-proliferative activity of cabozantinib on liver cancer cells is limited, and its anticancer effect is more likely due to its action on the tumor microenvironment. In the same investigation, it was also found that overexpression of NBAS and LAPTMA4 genes may result in resistance to cabozantinib (64).

Another study conducted by Rodríguez-Hernández MA et al. examined the potency of TKIs against liver cancer cells that have different cell differentiation stages and p53 expression (65). Cabozantinib was shown to be more cytotoxic on SNU423, SNU449, and Huh7 cells (poorly to moderately differentiated cells with deleted or mutated p53) than HepG2 and Hep3B cells (highly differentiated cells with wild-type or non-sense mutated p53), suggesting that cabozantinib was particularly effective on poorly differentiated HCC cell lines harboring mutation or lack of p53. These poorly differentiated cells were reported to have a lower basal oxygen consumption rate, ATP generation, and maximum respiratory capacity than well-differentiated HCC cells, implying the potential of cabozantinib to target the tumor microenvironment as reported by Caruso’s study (64, 65). Shang et al. also evaluated cabozantinib’s efficacy in a panel of 14 human HCC cell lines. The authors found that hyperphosphorylated/activated (p)-MET, total (t)-AXL, and p-ERK1/2 levels were inversely correlated with cabozantinib half-maximal inhibitory concentration (IC_50_) (66). Overall, all the cell line studies support the hypothesis that cabozantinib inhibits HCC growth mainly *via* the c-MET pathway.

To further illustrate the therapeutic efficacy of cabozantinib for HCC treatment, Shang et al. determined the effects of cabozantinib in four oncogene-driven murine HCC models, including the c-Met/β-Catenin (67), Akt/c-Met (68), Akt/Ras (69), and c-Myc (70) mice. Specifically, it was found that cabozantinib treatment led to stable disease in c-Met/β-catenin and Akt/c-Met mouse lesions, whereas it did not display efficacy in Akt/Ras and c-Myc mouse HCC models. Mechanistically, it was shown that in c-Met/β-catenin and Akt/c-Met HCC lesions, cabozantinib strongly inhibited p-Met levels, leading to decreased phosphorylated/activated (p)-Erk1/2 levels. In contrast, while c-Met protein expression could be detected in Akt/Ras and c-Myc mouse HCC, c-Met was not activated in these mouse HCCs, and p-Erk1/2 expression was not affected by cabozantinib administration. Interestingly, cabozantinib treatment resulted in the pronounced decreased expression of pyruvate kinase isozymes M2 (PKM2) in c-Met/β-catenin and Akt/c-Met HCC as well as human HCC cells. As PKM2 is a major enzyme regulating glycolysis, the results suggest that cabozantinib may affect HCC glycolysis. Another important conclusion from the study was that cabozantinib could modulate tumor microenvironment as it inhibited tumor angiogenesis *via* suppressing phosphorylated/activated (p-)VEGFR2 regardless of the oncogenic drivers. The inhibition of angiogenesis led to extensive tumor necrosis in c-Myc and Akt/Ras HCC models. On the other hand, cabozantinib treatment had a limited impact on cancer-associated fibroblasts, macrophages, and tumor-infiltrating T cells.

In summary, combining *in vitro* and *in vivo* studies, the study by Shang et al. indicates that cabozantinib might be effective against a subset of human HCCs with activated c-MET signaling *via* suppressing the c-MET/ERK pathway. Furthermore, cabozantinib is a potent angiogenesis inhibitor, although inhibition of angiogenesis alone may not be effective in arresting HCC progression.

## Cabozantinib Based Combination Therapies for HCC

Drug combination therapy is widely recognized to be more efficient in treating cancer than the administration of a single drug. Due to the somehow moderate efficacy of cabozantinib for cancer treatment, it has been suggested to combine cabozantinib with other medications for increased effectiveness and/or decreased toxicity. For instance, a mouse colon cancer cell line showed that cabozantinib, in combination with a poxviral-based cancer vaccine targeting a self-antigen, reduced Treg cells in the tumor and increased cytokine production by the effector T cells, leading to significantly increased anti-tumor activity (24).

The first preclinical cabozantinib-based combination therapy was reported by Wang et al. (71). They showed the combined use of cabozantinib with the novel focal adhesion kinase (FAK) inhibitor CT-707 in suppressing HCC. At the molecular level, the authors discovered that cabozantinib treatment in HCC cell lines leads to FAK activation (71). FAK is known to promote cell proliferation, survival, and migration and stimulate tumor angiogenesis (72). Thus, the compensatory activation of the FAK pathway might cripple the therapeutic efficacy of cabozantinib for HCC treatment. The authors applied CT-707, a FAK inhibitor, in combination with cabozantinib to test this hypothesis. The results suggested that cabozantinib and CT-707 synergized to suppress HCC growth *in vitro*. The combination therapy also demonstrated increased efficacy in a HepG2 xenograft model (71). Thus, the combined administration of cabozantinib and FAK inhibitors might be a promising strategy for treating HCC.

Additionally, Dong et al. reported that tumor-associated M2 macrophages could secrete HGF to make liver cancer cells resistant to sorafenib treatment (73). HGF secreted by M2 macrophages and tumor cells activated many growth factor cascades involved in tumorigenesis, such as HGF/c-MET, MAPK/ERK1/2, and PI3K/AKT pathways. HGF could also recruit and accumulate more tumor-associated M2 macrophages in tumors and thus increase the resistance to sorafenib treatment. A previous analysis of biomarkers in samples from phase 3 clinical trials of sorafenib treatment showed that patients with lower plasma HGF concentrations before treatment might have a higher survival rate, emphasizing that tumor-related M2 macrophages and HGF in tumors are of great importance in response to sorafenib therapy (74). These studies provide new insights for treating sorafenib resistance in HCC *via* a combination of sorafenib and HGF inhibitors. As a multikinase inhibitor with an effective inhibitory effect on c-MET, cabozantinib, in combination with sorafenib, might contribute to improved efficacy of first-line systemic therapy with sorafenib.

Recently, in the study by Shang et al., it was discovered that despite cabozantinib was effective in inhibiting activated c-MET dependent p-ERK levels, it failed to suppress AKT/mTOR pathway in HCC cells (66). Activated AKT/mTOR signaling is a pivotal pathway regulating HCC growth, survival, and metabolism (75). Therefore, targeting the mTOR pathway has been suggested as a potential therapeutic approach in many cancer types, including HCC (75). It is worth noting that clinical studies have demonstrated that mTOR inhibitors have limited efficacy against advanced HCC when used as monotherapy (76). Based on these data, it was hypothesized that combined cabozantinib and mTOR inhibitors might possess improved efficacy against HCC. To test this hypothesis, MLN0128, a pan-mTOR inhibitor targeting both mTORC1 and mTORC2 complexes, was used (66). In human HCC cell lines, cabozantinib and MLN0128 synergized to inhibit HCC cell growth. Subsequently, the authors tested the combination of cabozantinib and MLN0128 in the c-MET/β-catenin HCC model. MLN0128 treatment alone did not demonstrate any therapeutic efficacy. Cabozantinib, used as a single agent, led to stable disease. In striking contrast, the combination treatment resulted in remarkable tumor regression (66). At the cellular level, the combination treatment led to potent inhibition of tumor cell proliferation and elevated apoptosis, leading to large areas of necrosis within the tumor lesions. At the molecular level, the combination therapy effectively inhibited the expression of p-MET, p-ERK, p-AKT, and p-mTOR in HCC cells and the c-MET/β-catenin mouse HCC lesions. Taken together, these data indicate that the combination of cabozantinib and mTOR inhibitors might represent a novel and effective treatment for advanced HCC.

**Figure 3:**
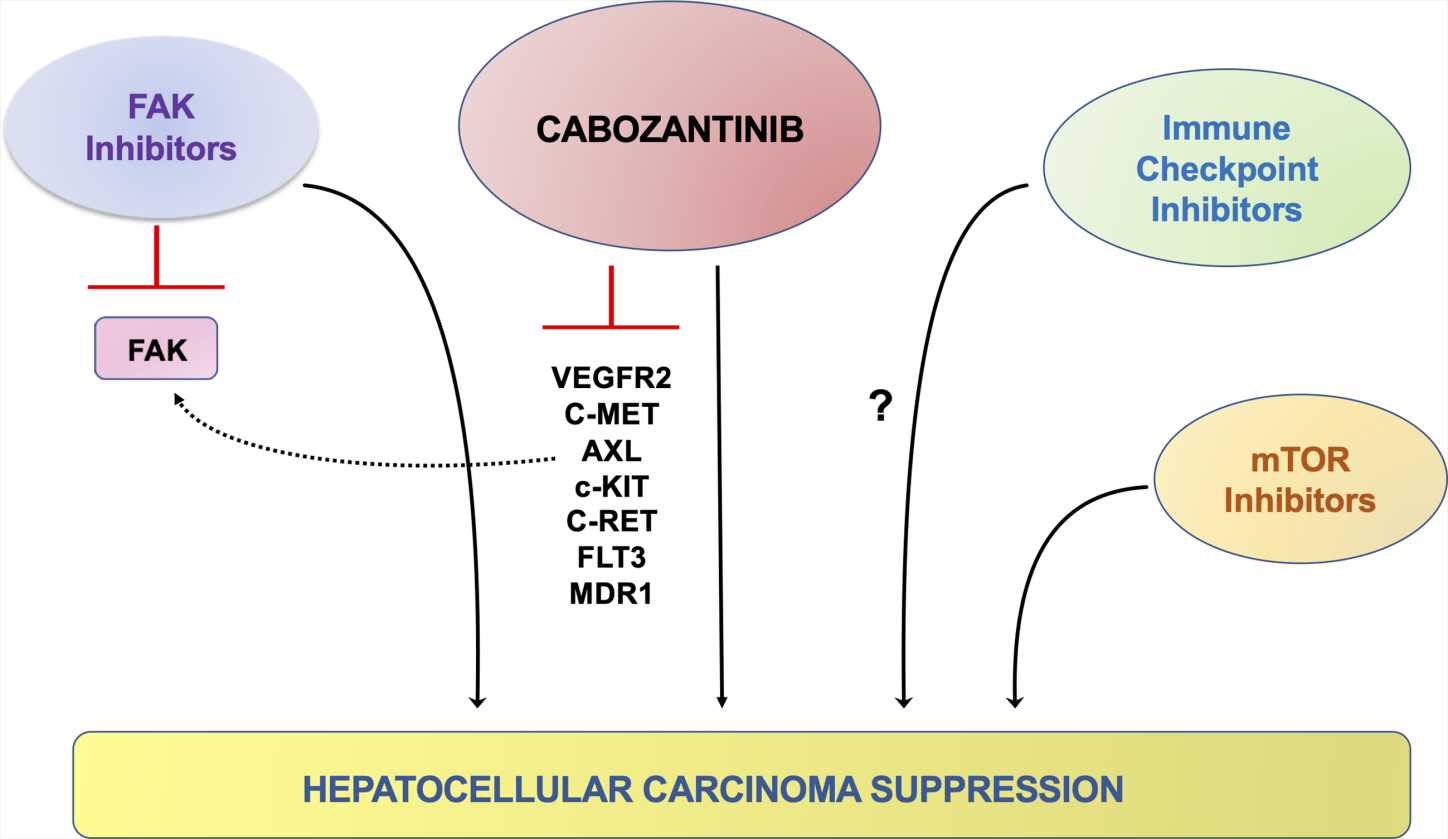
Schematic representation of the possible therapeutic strategies coupling cabozantinib to other signaling inhibitors in preclinical models of HCC. It has been previously shown that cabozantinib treatment on HCC cells leads to the compensatory activation (dotted, black line) of the FAK pathway, in which the use of FAK inhibitors can overcome. A synergistic, antineoplastic effect has also been observed when cabozantinib was co-administered to AKT/mTOR inhibitors. In addition, targeting the IGFR1 and FGFR1 pathways might hinder the resistance of cancer cells to cabozantinib administration. A possible synergistic effect could also be achieved with the concomitant treatment with cabozantinib and immune checkpoint inhibitors, although the data in this regard are contradictory (question mark).

Currently, clinical trials combining cabozantinib and immune checkpoint inhibitors, including PD-L1 (Trial ID: NCT03755791) and PD-1 (Trial ID: NCT04442581) for HCC treatment, are ongoing. The efficacy of cabozantinib and anti-PDL-1 antibody combination therapy was examined in the four mouse HCC models described above. Unfortunately, in contrast to a previous study suggesting that c-MET suppression leads to augmented PDL-1 expression in HCC (77), cabozantinib treatment did not increase PDL-1 levels in HCC cell lines and the mouse HCC models examined. Moreover, co-administration of cabozantinib and the anti-PDL-1 antibody did not lead to increased antineoplastic efficacy in the four murine HCC models examined (66). While the results do not provide preclinical support for the combination of cabozantinib and immune checkpoint inhibitors for HCC treatment, it is crucial to recognize that mouse models may not fully recapitulate the human disease, and additional studies are required to address this important issue. The possible therapeutic strategies of combining cabozantinib with other drugs are summarized in **Figure 3**.

## Cabozantinib and HCC: Obstacles

The use of cabozantinib in the clinic is frequently related to high-level adverse events. The grade 3 or 4 adverse events of cabozantinib are more common, such as hypertension, palmar-plantar erythrodysesthesia, diarrhea, fatigue, hand-foot syndrome, and increased aspartate aminotransferase level (13). In addition, six patients taking cabozantinib experienced each of the following grade 5 adverse events: liver failure, bronchoesophageal fistula, portal vein thrombosis, upper gastrointestinal bleeding, pulmonary embolism, and hepatorenal syndrome. Immune checkpoint inhibitors may be associated with immune-related side effects in the skin, intestine, thyroid, adrenal glands, lungs, or liver (78). A meta-analysis of randomized phase 2 and 3 immunotherapy trials demonstrated that immune checkpoint inhibitors were associated with all-grade colitis, elevated aspartate aminotransferase, skin rash, hypothyroidism, and pneumonia (79). Among them, the incidence of high-grade colitis and elevated aspartate aminotransferase levels was significantly higher. Therefore, when immune checkpoint inhibitors are used in combination with cabozantinib, diarrhea and elevated aspartate aminotransferase levels may be hard to avoid due to the same adverse events that appear in treatments with cabozantinib and immune checkpoint inhibitors.

The late diagnosis due to the lack of specific symptoms and reliable predictive biomarkers is often responsible for HCC’s poor prognosis. During the past decades, blood levels of the tumor marker serum alpha-fetoprotein and medical imaging have been the primary diagnostic methods for this disease. However, these tools are usually valuable for diagnosing HCC at the late stage. Therefore, people have made great efforts to discover biomarkers to identify the tumor in its early phases. Several novel biomarkers with high sensitivity and specificity, such as DCP, GPC3, and MDK, and biomarkers associated with poor outcomes, including p-ERK1/2, CD24, and MMP-12, have been identified (80). However, these biomarkers still require verification and recognition in HCC clinical trials and among different populations. In the SHARP phase 3 clinical trial, the plasma concentrations of angiopoietin 2 and VEGFA proteins were established as independent predictors for the survival of patients with advanced HCC. However, these biomarkers could not predict the prognosis of HCC patients treated with sorafenib, lenvatinib, or other oral medications (74, 81). The albumin-bilirubin levels can also predict the prognosis of patients with HCC, and a high level of bilirubin and a low level of albumin indicate a worse outcome (82). Compared with patients receiving placebo, patients treated with cabozantinib had more prolonged overall survival and progression-free survival, although these beneficial effects were not significantly related to the albumin-bilirubin levels (83). Thus, one future direction to help HCC treatment is to identify more predictive biomarkers for systemic therapy.

Cabozantinib was reported as a substrate of CYP3A4, a critical enzyme mainly found in the liver and intestine, and multidrug resistance protein 2 *in vitro* (84). After long-term use, cabozantinib would be expelled out of the cancer cells and contribute to the development of resistance when the expressions of CYP3A4 and multidrug resistance protein 2 increased. Therefore, finding an efficient CYP3A4 inhibitor or multidrug resistance protein 2 inhibitor may improve the systematic exposure of cabozantinib in cancer therapy under the premise of ensuring the safety of patients.

Besides the fact that cabozantinib is a substrate of ABC-transporters, it has been shown that the acquired resistance of cabozantinib would be developed with but not limited to the following mechanisms. First, in metastatic prostate cancer, some tumor cells still survived and expressed high levels of pFAK-Y397 and pTalin-S425, which are mediators of integrin signaling, after cabozantinib treatment (85). Using the FAK inhibitor PF-562271 to target integrin could inhibit FAK phosphorylation and reduce the survival rate of prostate cancer cells. Compared with the treatment with cabozantinib alone, the combination of PF-562271 and cabozantinib delayed tumor recurrence, suggesting that the activated integrin signaling pathway contributes to the resistance to cabozantinib. Second, Somwar et al. performed next-generation sequencing on tumor samples after cabozantinib treatment and found that *de novo* MDM2 amplification or TP53 mutations appeared in 50% of patients’ tumors (86). Acquired MDM2 amplification was detected in 50% of patients who relapsed after treatment with cabozantinib. When authors applied a combination treatment of cabozantinib and an MDM2 inhibitor, AMG232, on lung cancer, the synergistic therapy was shown to be more efficacious in suppressing lung cancer growth than monotherapy *in vitro* and *in vivo*, demonstrating that MDM2 amplification could be a potential mechanism of cabozantinib resistance in the clinic. Third, as reported by Varkaris et al. (87), cabozantinib can effectively inhibit tumor growth in the PDX models of prostate cancer and angiogenesis. However, residual tumor cells survived from cabozantinib treatment due to vascular heterogeneity represents a mechanism of cabozantinib resistance, and the initiation of the expression of FGFR1 in tumor cells is another potential mechanism of acquired resistance to cabozantinib (87). In the following study conducted by Koinis et al. (88), the authors showed that in prostate cancer, the inhibition of MET caused by cabozantinib treatment was through the activation of FGFR1. At the molecular level, FGFR1 was activated through a YAP/TBX5-dependent mechanism. Thus, YAP and its downstream target, TBX5, are critical factors in inducing the acquired resistance of cabozantinib. Furthermore, cabozantinib was demonstrated to inhibit the proliferation of osteoblasts *via* inhibiting VEGFR2 and MET in primary mouse osteoblasts, but it enhanced their differentiation (89). Subsequent mechanistic studies showed that during cabozantinib treatment, osteoblasts secreted a series of proteins, including pappalysin, IGFBP2, WNT 16, LEFTY1, and DKK1, which helped to inhibit the proliferation of osteoblasts and stimulate differentiation. The authors further found that these cabozantinib-induced secreted factors could contribute to cabozantinib resistance when treating the prostate cancer cells with the medium containing the secreted factors (89).

Last but not least, the Fuse group established several TKI-resistant cells, one of which was the cabozantinib-resistant KM12 cell line (90). The authors found that the activation of insulin growth factor receptor type 1 (IGFR1) was the primary mechanism causing the resistance to cabozantinib, and the combination of cabozantinib with an IGF1R inhibitor, such as OSI-906, could overcome cabozantinib resistance in KM12 cells. Even though the development of cabozantinib resistance has not been reported in HCC (at least from my search), the abovementioned mechanisms could also be a problem in the future treatment of HCC. Thus, more research on proper combination treatments to circumvent cabozantinib resistance needs attention.

## Cabozantinib and HCC: Future Directions

In cancer research, the use of predictive animal models for developing and evaluating the drugs at the preclinical level is of pivotal importance. Nonetheless, several studies showed that the anticancer activity of some agents in tumor models was not closely related to the effect in cancer patients, which questions the importance of preclinical models for the human disease. Although the results of at least one-third of the preclinical xenograft models have been validated in phase II clinical trials, the *in vivo* activity prediction of anticancer compounds using cell line-based traditional xenograft models for specific cancer types in the clinics is still poor (91). Noticeably, the cancer organoids or patient-derived tumor xenograft (PDX) models exhibit clinical specimens’ true tumor heterogeneity and genetic characteristics. Moreover, PDX models retain the histological and genetic features during the passages *in vitro*. Thus, they could be effectively used as a substitute model for the preclinical evaluation of new drugs (92). Thus, the use of cancer organoids or PDX models might be of significant help to validate preclinically the efficacy of novel targeted therapies against HCC.

In 2015, Xiang’s group reported that cabozantinib could overcome the multidrug resistance of HCC cells by inhibiting the function of multidrug resistance 1(MDR1)/P-glycoprotein (93). Thus, cabozantinib might be used in association with other chemotherapeutic agents whose potency is either completely hampered or reduced by P-glycoprotein overexpression. The treatment of HCC could also be improved by combining the administration of cabozantinib with immunotherapy, such as immune checkpoint inhibitors. Indeed, previous evidence indicates that the combination of targeted drugs (e.g., lenvatinib, bevacizumab) and PD-1 inhibitors (e.g., pembrolizumab, atezolizumab) is effective in ~30% of HCC at the clinical level (6, 94).

Although cabozantinib has many advantages for the treatment of HCC over other drugs, including the bioavailability, tolerable adverse events, and the ability to overcome P-glycoprotein overexpression, the efficacy of cabozantinib is often compromised by the development of drug resistance. Thus, additional studies are necessary to understand better the molecular mechanisms responsible for resistance to cabozantinib in HCC as well as to design therapeutic strategies aimed at preventing these compensatory events induced by HCC cells. In this regard, preliminary investigations support the use of either FAK (71) or mTOR inhibitors (66) in combination with cabozantinib for a more robust antineoplastic activity in HCC. Innovative approaches using high-throughput methodologies and additional investigations should be conducted to elucidate this crucial issue.

## Author Contributions

SD, AS, and DC drafted the manuscript. AS and DC prepared the figures. All authors contributed to the article and approved the submitted version.

## Funding

The salary of SD is supported by an National Institutes of Health grant R01CA190606.

## Conflict of Interest

The authors declare that the research was conducted in the absence of any commercial or financial relationships that could be construed as a potential conflict of interest.

## Publisher’s Note

All claims expressed in this article are solely those of the authors and do not necessarily represent those of their affiliated organizations, or those of the publisher, the editors and the reviewers. Any product that may be evaluated in this article, or claim that may be made by its manufacturer, is not guaranteed or endorsed by the publisher.

## Abbreviations

AXL, AXL receptor tyrosine kinase; CI, confidence interval; c-MET, MET proto-oncogene; FAK, focal adhesion kinase; FDA, U.S. Food and Drug Administration; FGFR, fibroblast growth factor receptor; FLT, fms like tyrosine kinase; HCC, hepatocellular carcinoma; HGF, hepatocyte growth factor; HR, hazard ratio; MAIC, matching-adjusted indirect comparison; ORR, objective response rate; PDGFR, platelet-derived growth factor receptor; PD-L, programmed cell death ligand; PDX, patient-derived tumor xenograft; PFS, progression-free survival; PI3K, phosphatidylinositol 3-kinase; RTK, receptor tyrosine kinases; TKI, tyrosine kinase inhibitor; VEGFR, vascular endothelial growth factor receptor.
